# Randomised controlled trial of a theory-based intervention to prompt front-line staff to take up the seasonal influenza vaccine

**DOI:** 10.1136/bmjqs-2019-009775

**Published:** 2019-08-05

**Authors:** Kelly Ann Schmidtke, Peter G Nightingale, Katharine Reeves, Suzy Gallier, Ivo Vlaev, Samuel I Watson, Richard J Lilford

**Affiliations:** 1 Department of Psychology, Manchester Metropolitan University, Manchester, Greater Manchester, UK; 2 Queen Elizabeth Hospital, University Hospitals Birmingham NHS Foundation Trust, Birmingham, UK; 3 Warwick Business School, University of Warwick, Coventry, West Midlands, UK; 4 Warwick Medical School, University of Warwick, Coventry, West Midlands, UK

**Keywords:** infection control, randomised controlled trial, health policy, communication

## Abstract

**Objective:**

To evaluate the effectiveness of reminder letters informed by social normative theory (a type of ‘nudge theory’) on uptake of seasonal influenza vaccination by front-line hospital staff.

**Design:**

Individually randomised controlled trial.

**Setting:**

A large acute care hospital in England.

**Participants:**

Front-line staff employed by the hospital (n=7540) were randomly allocated to one of four reminder types in a factorial design.

**Interventions:**

The standard letter included only general information directing the staff to take up the vaccine. A second letter highlighted a type of social norm based on peer comparisons. A third letter highlighted a type of social norm based on an appeal to authority. A fourth letter included a combination of the social norms.

**Main outcome measure:**

The proportion of hospital staff vaccinated on-site.

**Results:**

Vaccine coverage was 43% (812/1885) in the standard letter group, 43% (818/1885) in the descriptive norms group, 43% (814/1885) in the injunctive norms group and 43% (812/1885) in the combination group. There were no statistically significant effects of either norm or the interaction. The OR for the descriptive norms factor is 1.01 (0.89–1.15) in the absence of the injunctive norms factor and 1.00 (0.88–1.13) in its presence. The OR for the injunctive norms factor is 1.00 (0.88–1.14) in the absence of the descriptive norms factor and 0.99 (0.87–1.12) in its presence.

**Conclusions:**

We find no evidence that the uptake of the seasonal influenza vaccination is affected by reminders using social norms to motivate uptake.

## Introduction

Every year the National Health Service (NHS) hospitals and community services in England offer their front-line staff free, onsite influenza vaccination to protect patients from infection, and reduce staff absences in case of an epidemic. The NHS incentivises hospitals to maximise vaccination rates by providing payments against achievement of a threshold vaccination rate. In the 2017/2018 season the target level was 70%, and front-line staff vaccination rates reported across NHS trusts varied from 38.9% to 92.3% with the median trust achieving 70.8%.^1^ For the 2018/2019 season, NHS England offered a financial reward to trusts that met a 75% vaccination rate for front-line staff.^2^ To meet this stretching target, the University Hospitals Birmingham NHS Foundation Trust (UHB) approached the National Institute for Health Research (NIHR) Collaboration for Leadership in Applied Health Research and Care (CLAHRC) West Midlands for support in designing and evaluating a new intervention as part of their seasonal influenza vaccination campaign.

In coproducing the intervention, UHB stipulated that it needed to be suitable for scale and spread to other hospitals,^3^ not run counter to other measures within their planned vaccination campaign and be informed by behavioural science frameworks such as nudge theory.^4^ Based on these requirements, four letter-based interventions were developed. One letter was a standard reminder to recipients to have the vaccination while the others were based on one of two behavioural theories or on both. The theories both belong to a set of theories which make appeals to social norms. Social norms are tacit societal rules that guide how people believe they ought to interact with each other.^5^ The social norms we tested included two types: descriptive norms and injunctive norms.^6^ Descriptive norms are based on evidence that people are influenced by comparisons of their behaviour to that of their peers, for example, the bandwagon effect.^7^ Injunctive norms are based on evidence that people are influenced by the personalised appeals of authority figures.^8^

To compare the effectiveness of different norms, in isolation and combination, the staff were randomly allocated to receive one of four letters: a standard letter encouraging the staff to take up the vaccination (no norms), a letter appealing to descriptive norms, a letter appealing to injunctive norms and a letter that combines these norms. Our first objective was to assess the effects of the two social norms and any interaction effects on the on-site vaccination rate in a factorial-designed randomised controlled trial (RCT). Our hypothesis was that the letters would influence staff uptake differently. Our secondary objectives were to examine the differences between staff characteristics and the effects of the interventions on vaccine uptake rates.

## Methods

### Trial design

A randomised two-by-two factorial design was used to evaluate the effectiveness of four different letters reminding the staff to take up the seasonal influenza vaccine: standard letter, descriptive norms letter, injunctive norms letter and combination letter. The trial took place within one of the largest acute care hospitals in England. All front-line staff were eligible for the trial. The hospital’s existing information systems provided the necessary data to individually randomise participants in a stratified fashion, to print the letters, to address envelopes to participants’ work mail slots and to retrieve the outcome data for those staff members included in our study.

**Figure 1:**
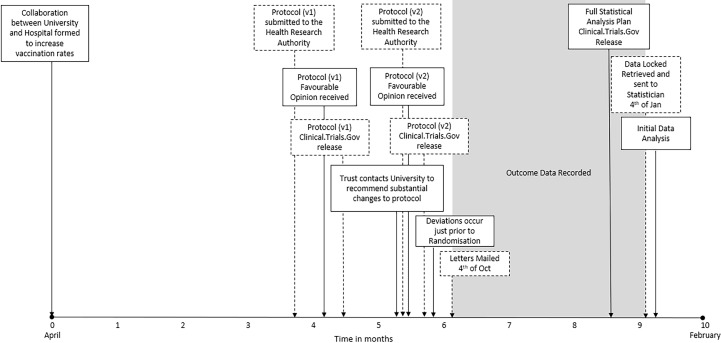
Timeline of events.

The host institution sponsored the trial. The trial was registered at ClinicalTrials.gov (ID: NCT03637036), where the protocol and statistical analysis plan can be viewed. A timeline describing the sequence of events is provided in figure 1.

### Staff included in the study and subgroups

The Hospital Electronic Staff Record was reviewed to identify front-line staff. The Electronic Staff Record includes staff ID, work mailing address, employment type (bank or substantive) and job type. Four job types comprise front-line staff according to the NHS incentive system: (1) medical and dental staff, (2) nursing, midwifery and health visiting staff, (3) scientific, therapeutic and technical staff, and (4) healthcare assistants and other support staff. Administration and estates, healthcare science and general payment staff were excluded. All other staff were included. This means that people with contraindications, who may have moved from the hospital soon after randomisation or who were vaccinated elsewhere, are included in the denominator, in accordance with intention-to-treat principles.

### Interventions and control condition

Front-line staff were allocated to one of four comparator groups: standard group, descriptive norms group, injunctive norms group and combination group. A copy of the letter sent to each group is provided in online supplementary material A. The standard letter reminded recipients that taking up the vaccination was important for patient and staff health, informed them that the expected national vaccination rate for front-line staff was at least 75% and directed them to find more information online or to ask their line manager. The descriptive norms letter included the contents of the standard letter and reported the vaccination rates at similar trusts in England and across all hospitals in the USA for the previous year. The injunctive norms letter included the contents of the standard letter, a personalised salutation and the signature of the Trust’s Chief Executive (who was previously the Medical Director) personally directing the staff to take up the vaccination. Participants allocated to the combination group received a letter containing the contents of the standard letter with the additions of both the descriptive norms and injunctive norms letters. Due to the nature of the intervention, the staff could not be blinded to their group but were not informed about the trial.

10.1136/bmjqs-2019-009775.supp1 Supplementary data

In addition to the letters, all staff were exposed to the Trust’s extensive staff influenza vaccination campaign. This campaign included ‘Communication methods’ and ‘Opportunity methods’ to influence staff behaviour. ‘Communication methods’ were designed to make the staff aware of the Trust’s intention that all staff should have the vaccination as a matter of patient and staff safety. ‘Communication methods’ also included information presented in regular staff emails/newsletters, face-to-face team briefings (grand round lectures and induction meetings for new staff), social media (Facebook and Twitter) and posters displayed physically around the hospital and digitally as computer screensavers. Online supplementary material B provides examples of these communications. ‘Opportunity methods’ included making the vaccination available within the clinical areas where staff regularly work, advertised vaccination clinics that staff could attend and at pop-up clinics in the hospital’s main entrance and other busy staff thoroughfares. The study was therefore designed to evaluate the marginal effect, if any, of adding ‘social norms methods’ to the standard ‘communication and opportunity methods’ used in the hospital.

10.1136/bmjqs-2019-009775.supp2 Supplementary data

### Outcomes

The study was prospective, but based on routinely collected data. The date staff members were vaccinated on-site was recorded on the Trust’s Flu-Jab database, along with their staff ID. The staff who were vaccinated off-site or refused the vaccination (say because of contraindications) could complete a notification form to be included in the database. While we recorded the number of staff who completed either of these forms, we did not analyse these data statistically because many staff who were vaccinated off-site or refused may have failed to fill out a form. The outcome data were not retrieved or reviewed by the research team until the data were ‘locked’ at 00:00 on 4 January 2019 (figure 1).

### Statistical power

At an alpha of 0.05, the sample size for the final study design (n=7540) had 90% power to detect a main effect of five percentage points from a 70% baseline and had 80% power to detect an interaction effect of six percentage points in the factorial design. No interim analysis took place.

### Randomisation and protocol deviations

Patients were randomised into groups using stratification by the above four job types. Two deviations from the randomisation process described in the protocol occurred. The ethical and methodological consequences of these deviations were determined non-substantial, as all staff received information by posters, email and word of mouth reminding them to be vaccinated (see the ‘Interventions and Control Condition’ section), statistical power remained sufficient for the planned analyses (see the ‘Statistical Power’ section) and all changes occurred prior to randomisation. Each deviation is described below:

1. While the registered protocol describes stratifying participants across four hospitals, three of which recently merged with UHB, only staff from the largest hospital took part, that is, Queen Elizabeth Hospital Birmingham. This deviation occurred because the research team was unable to reliably locate vaccination information from the three newly incorporated hospitals in the time available.
2. While the registered protocol describes randomising participants across four letter groups, a fifth group was created. This fifth group was a ‘no letter’ control group created without the University researchers’ awareness. This deviation occurred because the hospital managers decided that, for operational reasons, they needed to find out whether a letter of any type had an effect beyond that driven by the hospital’s existing vaccination campaign. The hospital decided that the no letter control group should be smaller than the other groups, but large enough to retain 80% power (alpha=0.05) to detect a difference of 5 in the percentage uptake for those staff allocated to the no letter control group and those allocated to receive any of the four letters. So, while each of the letter groups contained 1885 participants (22% of participants), the no letter control group contained 898 participants (11% of participants). The creation of this fifth group addresses the question of whether any letter might have produced an effect. We examine the possible effect of receiving no letter at all, separately from the prespecified factorial design.

After allocating participants on 24 September 2018, the information necessary to send participants their assigned letter was sent to the Trust’s mail department to print and send the letters. The letters were sent out on 4 October 2018 to coincide with the beginning of the Trust’s staff influenza vaccination campaign (figure 1).

### Statistical methods

The descriptive results (groupwise proportional uptake) were obtained across each group and subgroup along with the exact 95% binomial CIs.

Our primary analysis was a logistic regression analysis with on-site vaccination as the outcome variable and the two main intervention effects, descriptive norms and injunctive norms, and the interaction effect as explanatory variables.

A secondary analysis was conducted to explore the possible treatment effect of heterogeneity between subgroups. We ran a second logistic regression model that included interaction terms between the treatment effect indicators, job type and gender.

An additional secondary analysis was conducted to assess rates of uptake in each letter group over time. We anticipated that benefits of the interventions may be greater nearer to the beginning of the trial than nearer to the end, because the potential effects are likely to be most pronounced as the letters are delivered. Descriptively, this effect was initially assessed by visually examining Kaplan-Meier curves for cumulative vaccination rates, and CIs generated using the exponential Greenwood estimator.

Lastly, a separate analysis was conducted to compare the proportion of staff who were vaccinated in the no letter control group with those who received any of the four letters. This was analysed both with Fisher’s exact test and with a logistic regression analysis to predict on-site vaccination.

Data were analysed with SPSS Statistics for Windows V.22 (IBM).

### Patient and public involvement

There was no opportunity for patient and public involvement in this study.

## Results

**Figure 2:**
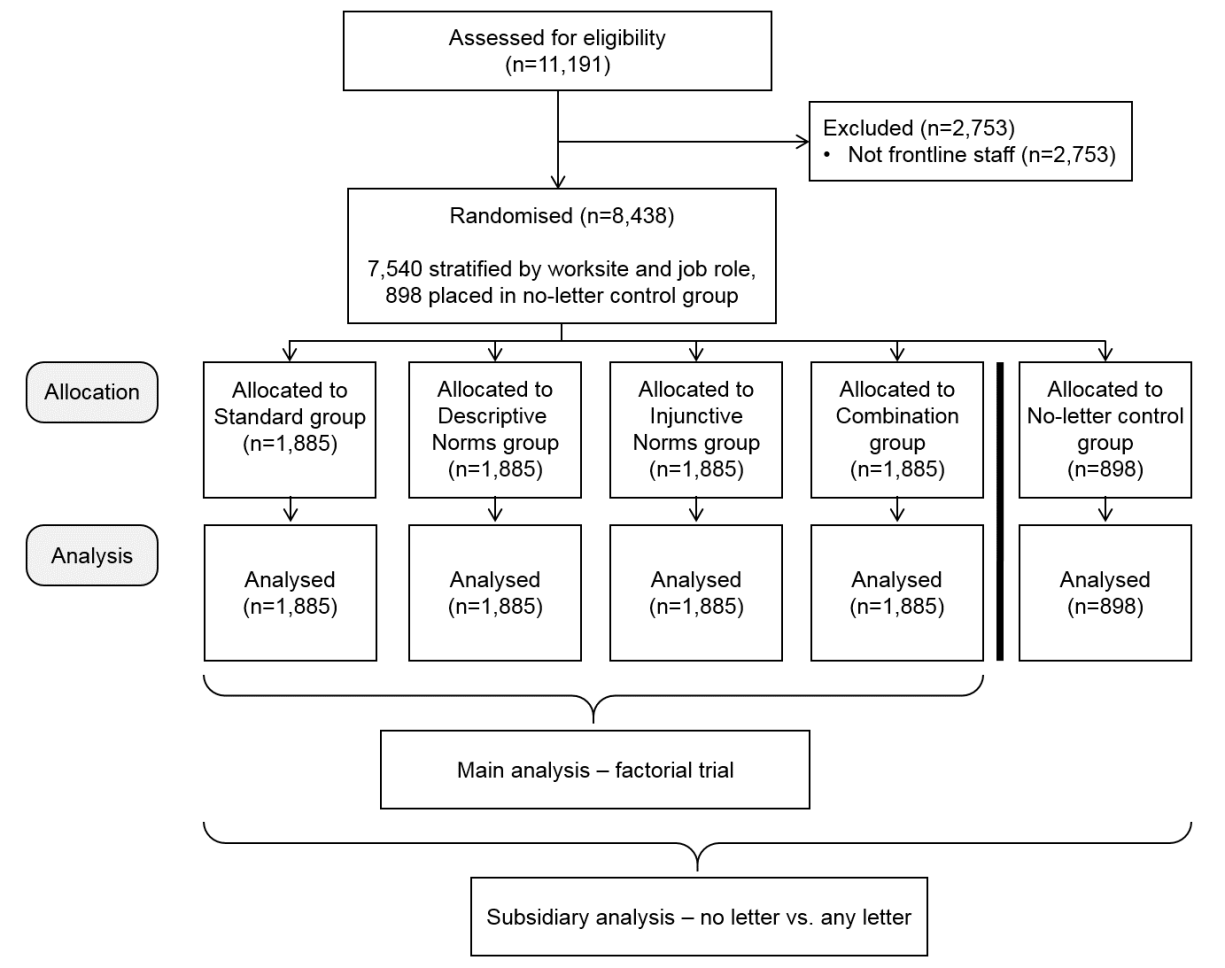
Participant flow chart.

Figure 2 presents a flow diagram describing the number of participants screened (n=11 191). Of these 11 191 participants, 2753 were excluded because the existing Trust records did not define them as being front-line staff. Of the remaining 8438 participants, 898 were allocated to the no letter control group, and 7540 participants were allocated to one of the four letter groups in an equal fashion, that is, 1885 in each letter group. Table 1 presents the number of participants by trial group. A very small number of staff may have left hospital employment over the study period, but any such attrition would have been non-informative.

**Table 1:** Baseline demographic data for each group. Percentages are given as percentages of the total number, that is, 7540

	All	Standard group	Descriptive norms group	Injunctive norms group	Combination group
Total	7540 (100.00%)	1885 (25.00%)	1885 (25.00%)	1885 (25.00%)	1885 (25.00%)
**Employment** **t** **ype**					
Bank	1280 (16.97%)	320 (4.24%)	320 (4.24%)	320 (4.24%)	320 (4.24%)
Substantive	6260 (83.02%)	1565 (20.76%)	1565 (20.76%)	1565 (20.76%)	1565 (20.76%)
**Job types**					
Medical and dental staff	1140 (15.12%)	285 (3.78%)	285 (3.78%)	285 (3.78%)	285 (3.78%)
Nursing, midwifery and health visiting staff	4380 (58.09%)	1095 (14.52%)	1095 (14.52%)	1095 (14.52%)	1095 (14.52%)
Healthcare assistants, other support staff*, Scientific, therapeutic and technical staff	2020 (26.79%)	505 (6.70%)	505 (6.70%)	505 (6.70%)	505 (6.70%)
Female	5600 (74.27%)	1392 (18.46%)	1415 (18.77%)	1390 (18.44%)	1403 (18.71%)

*We merged groups 3 and 4 into this group.

All trial groups demonstrated a similar vaccination uptake of approximately 43%. Table 2 presents the proportion of participants who took up the vaccination in each group overall and then by gender and for each stratified characteristic.

**Table 2:** Impact of each letter on staff uptake of the seasonal influenza vaccination. Exact 95% binomial CIs are presented in parentheses below the uptake proportion

	Standard group		Descriptive norms group		Injunctive norms group		Combination group	
	**n**	**Uptake proportion** (**95% CI**)	**n**	**Uptake proportion** (**95% CI**)	**n**	**Uptake proportion** (**95% CI**)	**n**	**Uptake proportion** (**95% CI**)
Total	1885	0.43 (0.41 to 0.45)	1885	0.43 (0.41 to 0.46)	1885	0.43 (0.41 to 0.45)	1885	0.43 (0.41 to 0.45)
**Gender**								
Female	1392	0.43 (0.40 to 0.46)	1415	0.43 (0.40 to 0.46)	1390	0.44 (0.41 to 0.46)	1403	0.44 (0.41 to 0.46)
Male	493	0.43 (0.39 to 0.48)	470	0.45 (0.40 to 0.49)	495	0.42 (0.37 to 0.46)	482	0.41 (0.37 to 0.46)
**Employment** **t** **ype**								
Bank	320	0.26 (0.22 to 0.31)	320	0.23 (0.19 to 0.28)	320	0.24 (0.20 to 0.29)	320	0.23 (0.18 to 0.28)
Substantive	1565	0.47 (0.44 to 0.49)	1565	0.48 (0.45 to 0.50)	1565	0.47 (0.45 to 0.50)	1565	0.47 (0.45 to 0.50)
**Job types**								
Medical and dental staff	285	0.46 (0.40 to 0.52)	285	0.46 (0.40 to 0.52)	285	0.46 (0.40 to 0.52)	285	0.47 (0.41 to 0.53)
Nursing, midwifery and health visiting staff	1095	0.45 (0.43 to 0.48)	1095	0.43 (0.40 to 0.46)	1095	0.45 (0.42 to 0.48)	1095	0.43 (0.40 to 0.46)
Healthcare assistants, other support staff, Scientific, therapeutic and technical staff	505	0.36 (0.32 to 0.40)	505	0.43 (0.39 to 0.47)	505	0.38 (0.34 to 0.43)	505	0.41 (0.37 to 0.45)

The results presented in table 2 consider only participants to be vaccinated if they were vaccinated on-site. Of the 151 participants who reported being vaccinated off-site (2% of the 7540 participants) 44 received the standard letter, 39 received the descriptive norms letter, 36 received the injunctive norms letter and 32 received the combined letter. Of the 541 participants who refused the vaccination (7% of the 7540 participants), 120 received the standard letter, 119 received the descriptive norms letter, 139 received the injunctive norms letter and 163 received the combined letter.

**Figure 3:**
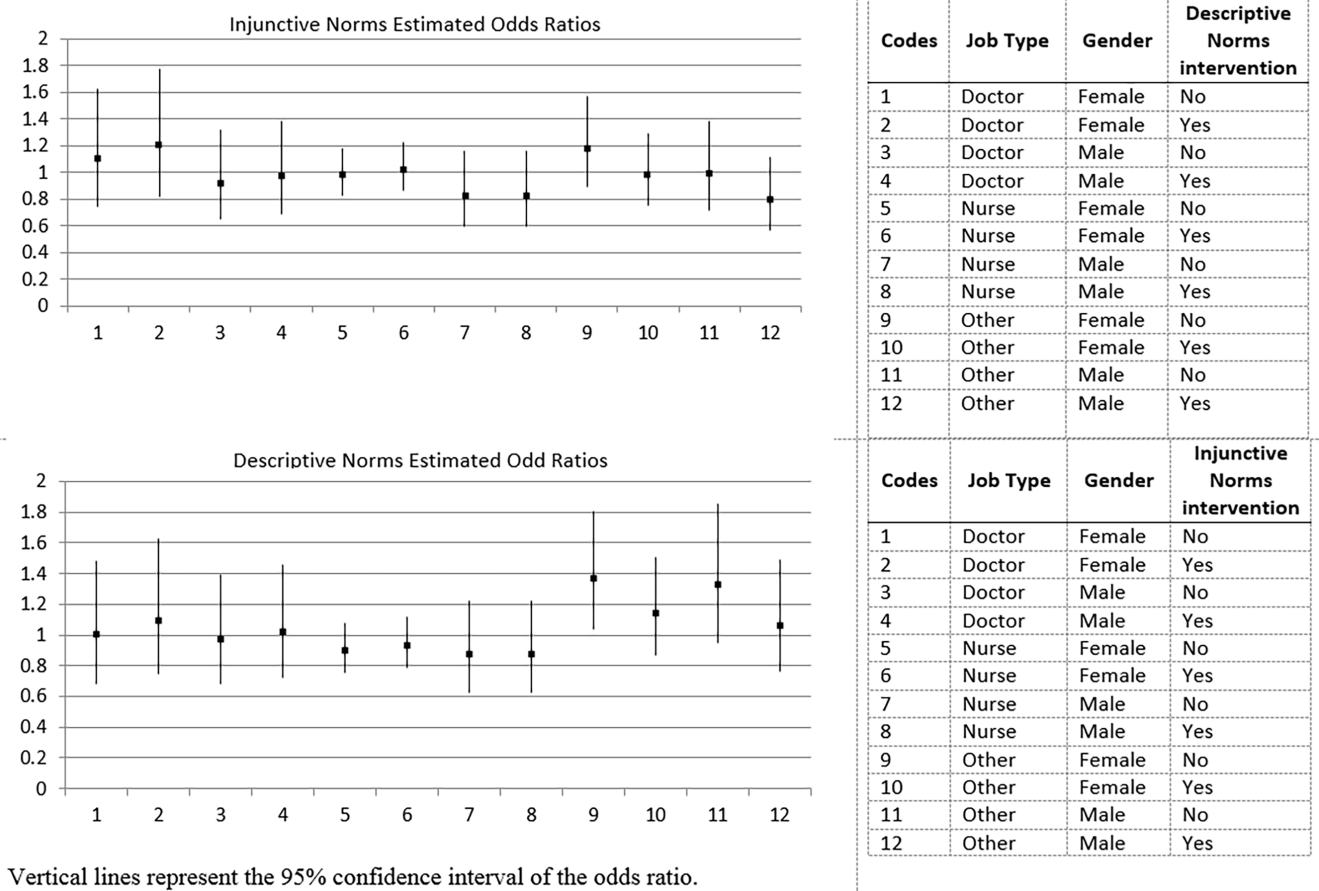
The estimated ORs from the subgroup analysis.

The primary analysis did not find any evidence for an effect of either social norm or their interaction. For on-site vaccination, the OR for the descriptive norms factor was 1.01 (0.89–1.15) in the absence of the injunctive norms factor and 1.00 (0.88–1.13) in its presence. The OR for the injunctive norms factor was 1.00 (0.88–1.14) in the absence of the descriptive norms factor and 0.99 (0.87–1.12) in its presence. Figure 3 presents the estimated ORs from the subgroup analysis. Only one of the 24 was statistically significant, although it was no longer so after correcting for multiple testing. No interactions were clinically significant.

**Figure 4:**
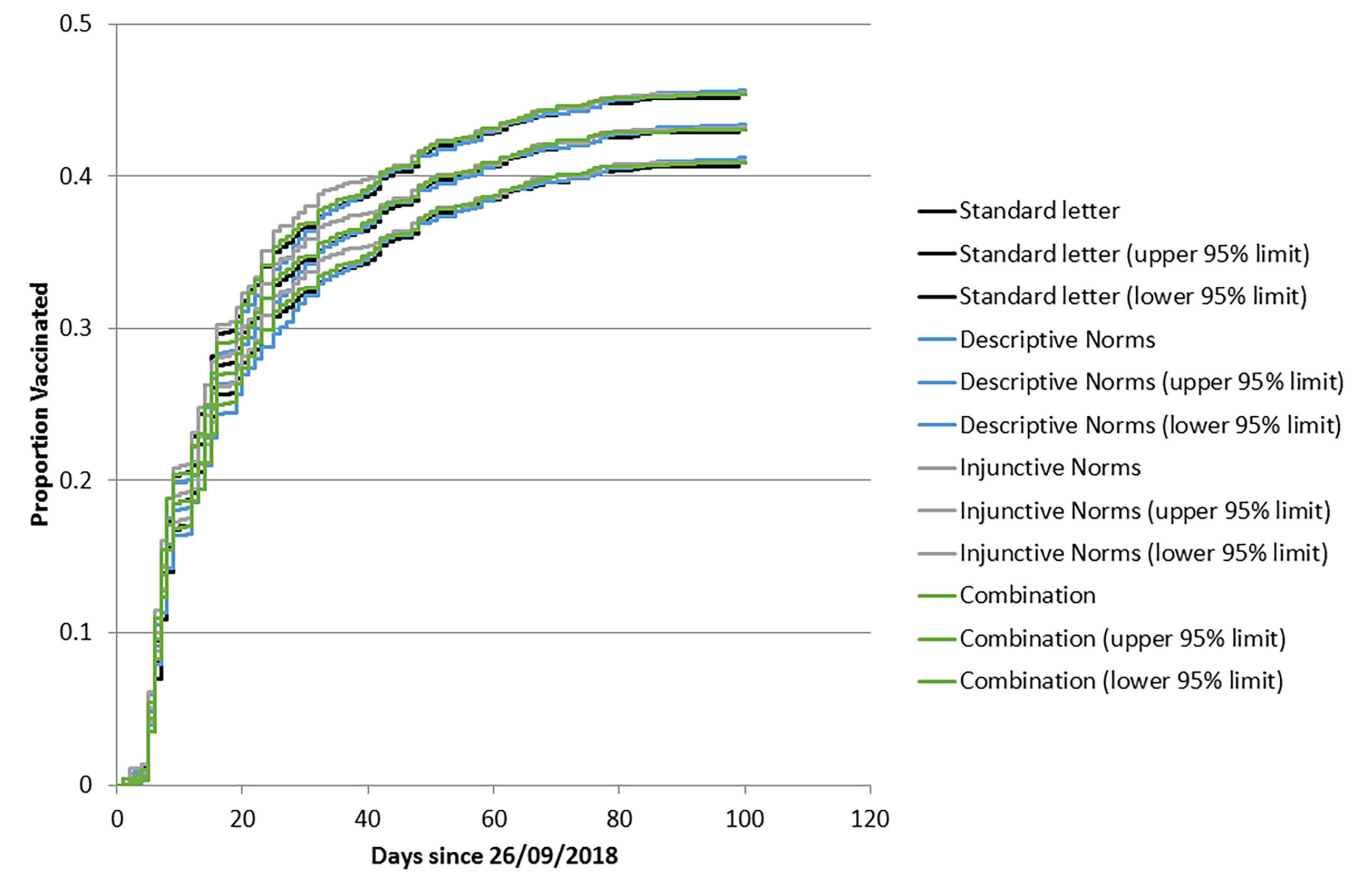
Survival plot of vaccinations by week for each letter group.

Kaplan-Meier curves showing cumulative vaccination rates are displayed in figure 4. The time it took for each group to reach a 40% vaccination rate on-site was similar, with the standard letter group doing so in 56 days (95% CI 43 to 75), the descriptive norms group in 56 days (95% CI 42 to 75), the injunctive norms group in 51 days (95% CI 42 to 72) and in 51 days (95% CI 42 to 70) for the combination group.

In the separate analysis to examine the effect of receiving any letter we found that the rate of vaccination in the no letter control group was 45% compared with 43% for all the participants who were sent a letter. This difference was not statistically significant.

## Discussion

### Principal findings

The trial finds no evidence supporting the effectiveness of reminder letters based on social norms theory on staff uptake of influenza vaccination. Uptake did not differ by occupational group and there was no consistent evidence of an interaction between occupational group and intervention. In addition, the current trial finds no support for the effectiveness of adding a reminder letter to an existing vaccination campaign, as there was no difference in uptake for participants who were sent a letter and participants in the no letter control group. This is in contrast to a previous trial that found that simply receiving a letter influenced vaccine uptake rates among the general public although the effect size was small (<1%).^9^ The hospital was already using several methods to maximise vaccination rates. These methods are supported by empirical evidence^4^ and psychological theories such as the Theory of Planned Behavior,^10^ the Health Belief Model,^11^ the Risk Perception Attitude Framework^12^ and the Triandis Model of Interpersonal Behavior.^4 13^ However, we find no marginal increase in vaccine uptake resulting from our theory-based intervention, notwithstanding the considerable headroom for further improvement.

### Findings in relation to previous studies to increase vaccination rates among hospital staff

A 2012 systematic review of studies undertaken to increase hospital staff influenza vaccination rates included 25 studies, only one of which was an RCT.^14^ That trial had a factorial design similar to the trial reported here, where staff received nothing (the control group), a letter, a raffle ticket or both a letter and a raffle ticket. They found a trend towards higher vaccine uptake, from 38% for staff in the control group to 44.5% for staff that received both a letter and a raffle ticket. This was not significant, but the study was an order of magnitude smaller (n=800) than that reported here.^15^

The above previous trial reported uptake rates very similar to the 43%–45% reported in our study. Nevertheless, the overall uptake reported here is lower than the reported national median and than the results reported in the study hospital. The overall vaccination rate reported in our trial does not match the hospital returns to NHS England due to different inclusion criteria. For example, students who work on the wards were included in the hospital returns, but we included only staff employed in the hospital, as recorded in the Electronic Staff Register, at the time of randomisation. We manually checked to ensure that each person in our numerator is included in the denominator. Differences in output between routine hospital data and academic studies have been the subject of an extensive literature.^16–19^

### Findings in relation to previous studies of letters containing nudge messages

Our findings add to a growing literature about ‘nudge-type’ behaviour change interventions. While it might seem surprising that human behaviour can be materially influenced by messages conveyed in a letter, the empirical literature shows that this is precisely what might happen. Witness, for example, the positive effect of a letter encouraging students from disadvantaged backgrounds to apply to ‘top’ universities.^20^ Likewise, reminder letters that notified university staff that they had been scheduled for a ‘flu shot’ appointment (specifying day, time and location) resulted in greater uptake rates (45%) than letters simply reminding the staff to set an appointment themselves (33%).^21^ A later study suggests that this effect may extend to healthcare workers though the trial likely contained too few participants (61 per group) to detect statistical effects.^22^ A further trial found that including social norm messages in standard reminder letters increased payment rates for overdue tax; the magnitude of effect varied across letter types from one to five percentage points.^23^ Displaying a ‘poster sized commitment letter’ outside a family doctor’s surgery resulted in a 19% reduction in inappropriate antibiotic prescribing according to a cluster RCT of doctors.^24^ On the other hand, a randomised trial of 228 000 Medicare beneficiaries found ‘no difference in vaccination rates across the four different letters tailored with behavioral science techniques.’^9^

There is a risk, however, that the literature is skewed towards positive results. Publication bias is a substantial risk in service delivery research generally,^25–28^ and a null result for an inexpensive and easily implemented intervention may seem anodyne while, if such a finding were positive, it may seem more newsworthy for being surprising and for having scalable implications for a change in practice.

### Strengths and weaknesses

Our study was considerably larger than any RCT of psychological interventions to increase staff uptake of influenza vaccine and had considerable statistical power to test, not just for the individual effects of the two intervention types, but for an interaction between them.^14^ The observed base rate implied a larger sample size. Using a baseline of 43% instead of 70%, the power for the main effect of five percentage points is 85% and for the interaction effect of six percentage points is 73% (the power to detect an interaction effect of seven percentage points is 85%). Thus, statistical power was not heavily degraded and the evidence points to a very small or no effect of the ‘nudges’ such that it is unlikely that the conclusion would be affected by an even larger sample.

A limitation of our study is the fact that staff were not blinded to their intervention group. Even though staff were not informed of the trial, some staff may have compared letters with colleagues. This raises the possibility that contamination of intervention groups by controls diluted the effect in the intervention. However, a high proportion of people would have needed to share letters, and the effect among those who shared would have had to be large, arguably implausibly large, to yield the almost identical results observed. The reverse, that the control group was activated by the intervention group, is unlikely for the above reason and because vaccine uptake in the control group was even lower than in the no letter group.

### Future research

The question arises as to what should be done in the future given that, within narrow confidence limits, the ‘nudge theory’ methods used here were not effective. An intervention focused on communities within hospitals, such as emergency care or operating theatre staff, may be more effective. Provision of default appointments has proven effective in various settings, but this may suit staff based in a particular place, rather than those, such as doctors, who work across many hospital locations.^21 22^ Alternatively a future intervention might draw on the success found for telephone-based interventions (texts and apps) in non-health worker populations.^29–31^ In this trial, over 80% of people who were vaccinated received their vaccine within the first 30 days of the campaign. Therefore, future interventions could focus on staff who remain unvaccinated after 1 month. Lastly, staff might be coerced, for example, by requiring unvaccinated staff to wear face protection masks when working with patients during the influenza season, as in many hospitals in the USA.^32^ The latter option infringes on personal liberty, but this might be justified by third party effects.

### Rapid response research

The current study is an example of a demand-led or ‘rapid response’ evaluation (*paper under submission*). The hospital approached the academic team in April 2018, less than 5 months before the intervention was needed. This meant that there was no time to raise grant funding and that the administrative/ethical approvals needed to be expedited. We were able to meet this exacting timetable because of the support provided by UHB to administer on-ground activities and data analysis, and because NIHR CLAHRC West Midlands had standing capacity for research design and for seeking the necessary approvals. As hospitals around the world adopt more comprehensive data systems, the opportunity for more rapid response studies is likely to increase. In order to make the best use of these opportunities it may be cost-effective for healthcare organisations to acquire more in-house analytic capacity. That said, meeting service and research imperatives is a challenge, as witnessed by deviations in the protocol between registration and launch of the study. Rapid response RCTs occupy an intermediate position between typical researcher-led protocols and fully retrospective analysis of data generated when policymakers use a lottery to determine access to interventions, as in the New Zealand migration experiment,^33^ and the Oregon study of health insurance.^34^

## Conclusion

Developing interventions to increase vaccination rates will likely remain a priority issue for many years. While the current study’s intervention was not effective, the study itself demonstrates a method through which new interventions can be quickly developed and evaluated, that is, rapid response evaluations.
