# Supplementary material for: Randomised controlled trial of a theory-based intervention to prompt front-line staff to take up the seasonal influenza vaccine
Source: BMJ Qual Saf. 2019 Aug 5;29(3):189–97. doi: 10.1136/bmjqs-2019-009775 (PMC7061920; doi:10.1136/bmjqs-2019-009775)
Supplement: Supplementary data [file bmjqs-2019-009775supp001.pdf]

Supplementary Materials A. The four reminder letters, one on each of the next four pages. For illustrative purposes, the active parts of the Descriptive Norms factor are highlighted in yellow and the active parts of the Injunctive Norms factor are highlighted in green. The letters sent to staff did not contain yellow or green highlights.

University Hospitals Birmingham
NHS Foundation Trust

Queen Elizabeth Hospital Birmingham
Mindelsohn Way
Edgbaston
Birmingham B15 2GW

Tel: 0121 627 2000

October 2018

Dear colleague,

You are invited to take up your free flu jab this season to reduce your risk, and your patients' risk, of getting the flu.

Last winter, the national expectation was for at least 70% of frontline staff to be vaccinated against seasonal flu.

This year, the national expectation is for at least 75% of frontline staff to be vaccinated.

To find out how to get your vaccination at the Trust, please visit www.uhb.nhs.uk/flu or ask your department's senior sister or charge nurse.

We appreciate your support in keeping colleagues and patients safe.

Yours faithfully,

Occupational Health Department

Chair: Rt Hon Jacqui Smith Chief Executive: Dr David Rosser

University Hospitals Birmingham
NHS Foundation Trust

Queen Elizabeth Hospital Birmingham
Mindelsohn Way
Edgbaston
Birmingham B15 2GW

Tel: 0121 627 2000

October 2018

Dear colleague,

You are invited to take up your free flu jab this season to reduce your risk, and your patients' risk, of getting the flu.

Last winter, the national expectation was for at least 70% of frontline staff to be vaccinated against seasonal flu. That was achieved at QEHB, with 71.6%, and at Heartlands, Good Hope and Solihull with 73.5%.

This year, the national expectation is for at least 75% of frontline staff to be vaccinated.

However, we want UHB to be amongst the very best performers of similar hospitals, so that we maximise protection for our staff and patients.

We know it is entirely achievable, with your support, for us to reach or surpass the levels of some of the best performers from last year:

Royal Liverpool and Broadgreen	86.9% (of frontline staff vaccinated)
Cambridge University Hospitals	84.3%
Birmingham Children's Hospital	82.1%
Leeds Teaching Hospitals	80.8%
Guy's and St Thomas's	79.9%
In the United States, 92% of hospital staff received their free flu vaccination	

To find out how to get your vaccination at the Trust, please visit www.uhb.nhs.uk/flu or ask your department's senior sister or charge nurse.

We appreciate your support in keeping colleagues and patients safe.

Yours faithfully,

Occupational Health Department

Chair: Rt Hon Jacqui Smith Chief Executive: Dr David Rosser

University Hospitals Birmingham
NHS Foundation Trust

Queen Elizabeth Hospital Birmingham
Mindelsohn Way
Edgbaston
Birmingham B15 2GW

Tel: 0121 627 2000

October 2018

<First name> <Surname>

<Location>

Dear <First Name>

I am inviting you to take up your free flu jab this season to reduce your risk, and your patients' risk, of getting the flu.

Last winter, the national expectation was for at least 70% of frontline staff to be vaccinated against seasonal flu.

This year, the national expectation is for at least 75% of frontline staff to be vaccinated.

To find out how to get your vaccination at the Trust, please visit www.uhb.nhs.uk/flu or ask your department's senior sister or charge nurse.

I appreciate your support in keeping colleagues and patients safe.

Yours sincerely,

Dr David Rosser

Chief Executive

Chair: Rt Hon Jacqui Smith Chief Executive: Dr David Rosser

NHS
University Hospitals Birmingham
NHS Foundation Trust

Queen Elizabeth Hospital Birmingham
Mindelsohn Way
Edgbaston
Birmingham B15 2GW

Tel: 0121 627 2000

October 2018

<First name> <Surname>

<Location>

Dear <First Name>

I am inviting you to take up your free flu jab this season to reduce your risk, and your patients' risk, of getting the flu.

Last winter, the national expectation was for at least 70% of frontline staff to be vaccinated against seasonal flu. That was achieved at QEHB, with 71.6%, and at Heartlands, Good Hope and Solihull with 73.5%.

This year, the national expectation is for at least 75% of frontline staff to be vaccinated.

However, we want UHB to be amongst the very best performers of similar hospitals, so that we maximise protection for our staff and patients.

We know it is entirely achievable, with your support, for us to reach or surpass the levels of some of the best performers from last year:

**Royal Liverpool and Broadgreen**	86.9% (of frontline staff vaccinated)
**Cambridge University Hospitals**	84.3%
**Birmingham Children's Hospital**	82.1%
**Leeds Teaching Hospitals**	80.8%
**Guy's and St Thomas's**	79.9%
In the United States, c.92% of hospital staff receive their free flu vaccination	

To find out how to get your vaccination at the Trust, please visit www.uhb.nhs.uk/flu or ask your department's senior sister or charge nurse.

I appreciate your support in keeping colleagues and patients safe.

Yours sincerely,

Dr David Rosser

Chief Executive

Chair: Rt Hon Jacqui Smith Chief Executive: Dr David Rosser
