# Supplementary material for: Randomised controlled trial of a theory-based intervention to prompt front-line staff to take up the seasonal influenza vaccine
Source: BMJ Qual Saf. 2019 Aug 5;29(3):189–97. doi: 10.1136/bmjqs-2019-009775 (PMC7061920; doi:10.1136/bmjqs-2019-009775)
Supplement: Supplementary data [file bmjqs-2019-009775supp002.pdf]

Supplementary Materials B. Examples from the Trust's existing campaign.

1) Below is a picture of the front page of the Trust's campaign website. Clicking on the 'Get Vaccinated' button leads the user to a list of the Trust's vaccination clinic locations and times.

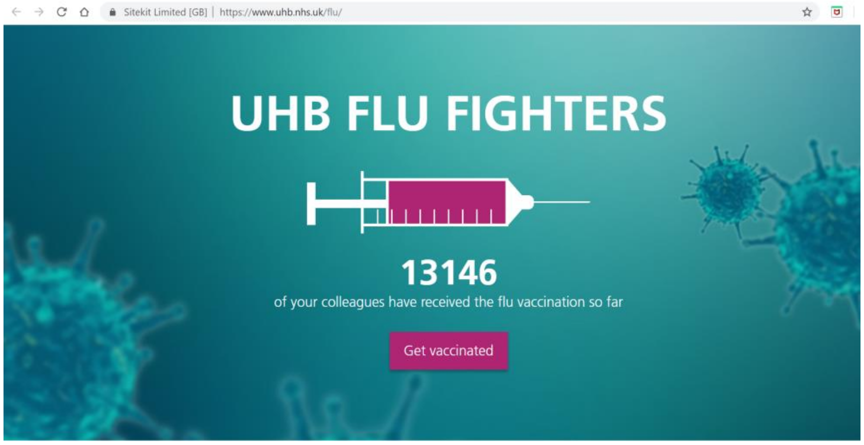

2) Below is an example of a message disseminated in the Trust's weekly e-bulletin, called 'In the Loop.'

Last winter was difficult

Winter is always a challenging time for the NHS. Last winter saw outbreaks of flu, respiratory and gastrointestinal illnesses that placed additional pressure on all departments, impacting our morale and performance.

The winter was longer and colder than recent years with over 290,000 more people attending emergency departments across the country.

The flu outbreak also proved to be the most severe in almost ten years with more than 500 patients per week admitted to hospitals at the peak of the season and a noticed increase in mortality amongst older patients.

We can reduce the risk of that happening again – helping to take some of that winter pressure off.

Many people with flu display no symptoms and unvaccinated staff are at risk of passing the virus on to more vulnerable patients, even where they themselves continue to feel well.

The best way to protect ourselves, families and patients is to have the flu vaccination. It will be available across all wards and departments from October 1.

See the new UHB flu fighter website from late September for details of the new quadrivalent flu vaccine – which offers the highest level of protection of any flu vaccination yet.

---

3) Below is a picture of one of the screensavers the Trust displayed during their campaign.

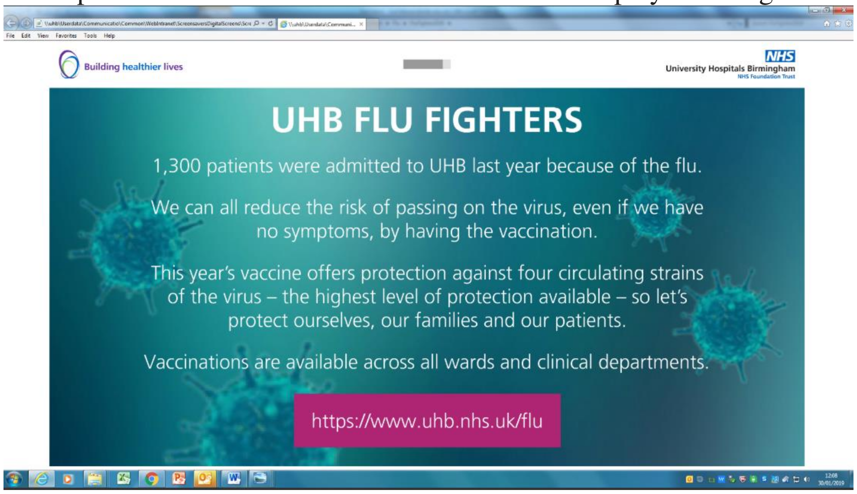

4) This is the web link to materials the NHS provides all trusts to use as part of their local vaccination campaigns: https://www.nhsemployers.org/campaigns/flu-fighter/nhs-flu-fighter/free-resources/posters-and-leaflets. Below is a picture of one of the posters.

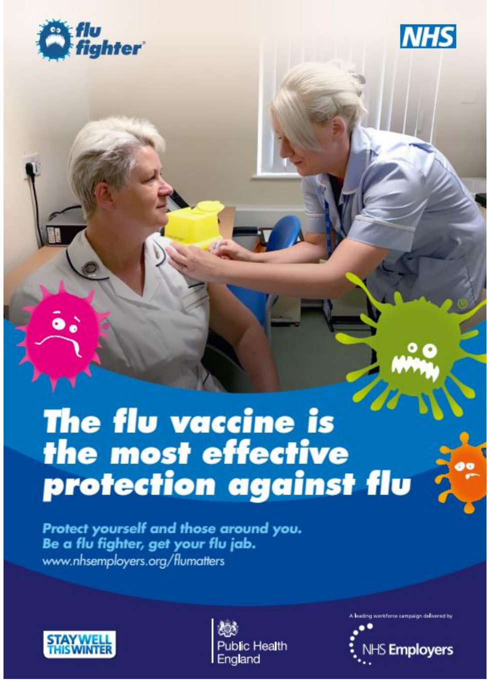

5) This is the web link to one of the Trust's initial campaign video https://vimeo.com/292338874/530c35688c. This video was posted on the 28th of September 2018 and has over 8,000 views as of January 2019. The video presents the director of nursing talking about how she originally avoided the flu vaccination for unscientific reasons and then changed her mind. She describes two main reasons she changed her mind: 1) she is a senior staff leader- and wanted to make sure she works as part of a team to protect patients, and 2) on a personal level, her mother had breast cancer and the thought that she would transfer an illness that could kill her mother made her believe that not getting the vaccination would be selfish. She then goes on to state the Trust's goal to vaccinate as near to 100% of staff as possible.
